# Are environmental damage and export concentration the major threats for the long-run economic growth in Bangladesh?

**DOI:** 10.1371/journal.pone.0284620

**Published:** 2023-04-25

**Authors:** Mohammad Mafizur Rahman, Eswaran Velayutham, Mohammad Abul Kashem

**Affiliations:** 1 School of Business, University of Southern Queensland, Toowoomba, Australia; 2 Bangladesh Bank, Dhaka, Bangladesh; University of Pitesti, Romania; Institute of Doctoral and Post-doctoral Studies, University Lucian Blaga of Sibiu, ROMANIA

## Abstract

Using World Bank and IMF data over the period 1990–2018, this research explores the determinants of economic growth in Bangladesh with particular attention to environmental degradation and export concentration variables. An ARDL (Autoregressive Distributed Lag) bound testing approach is employed as an estimation strategy with FMOLS (Fully Modified Ordinary Least Squares) and CCR (Canonical Cointegrating Regression) for cross-checking the results. The obtained results confirm that CO_2_ emissions, consumption expenditure, export concentration, remittances, and inflation are the main driving forces for the long-run economic growth in Bangladesh where the effects of the first two variables and the last three variables are positive and negative, respectively. The study also reveals the dynamic short-run relationships among the chosen variables. Environmental pollution and export concentration are found to be the barriers to economic growth; therefore, the country should take necessary steps to mitigate this problem for ensuring long-run sustainable economic development.

## 1. Introduction

Sustainable economic growth is the main policy target for any country regardless of development status because growth can help to achieve some of the most desirable macroeconomic objectives such as poverty reduction, increasing employment, improving public services, reducing the national debt and enhancing the overall well-being of people. Growth is particularly important in developing countries where poverty and unemployment problems are severe, investment and infrastructural development are low, the export base is narrow and the pollution level is high. Theoretically, economic growth trickles downs to the unemployed and poverty-stricken sections of society and increases the living standards of people, and thus economic growth, employment generation and poverty alleviation are highly interconnected. With economic growth, increased government tax revenue is collected that enables governments to spend more on public services; overall consumption and investment levels increase; life expectancy improves; productive economic activities gain momentum through high private and public investments. Governments are capable of reducing the debt-GDP ratio because of the solid foundation of the economy. Moreover, higher economic growth can allow governments to increase spending to protect environmental quality; it can also allocate resources for increased investment in research and development, which will ensure the sustainability of long-run economic growth. Economic growth is also linked to human development indicators such as freedom of choice, people’s skills, and capacities [1]. Thus, for every nation, economic growth is a prerequisite for the betterment of the country. However, economic growth may also lead to some negative effects such as income inequality (if the benefits of growth go to the rich more rather than the poor) [2], current account deficit (via more expenditure on imports) [3], inflation (because of rising aggregate demand) [4], and increased pollution (because of higher industrial production using non-renewable resources) [5, 6].

In the past, researchers have tried to identify growth factors using theory and empirical data across countries and regions. In general, empirical studies are conducted based on basic neoclassical growth theory as well as endogenous growth theory [7, 8] where capital and labour are used as the factors of production. According to Solow [9] and Grossman and Helpman [10], technological growth is essential for long-run economic growth. Other contributory factors to growth are social infrastructure, good governance and macroeconomic stability [11]. Empirically, researchers tried to identify the growth factors using different variables such as financial development, FDI, inflation [12], public expenditure [8], trade [13–15], energy [8, 16–19], population growth [14], remittances [8], and CO_2_ emissions [15, 20]. However, findings are inconclusive due to the heterogeneous characteristics of individual countries (e.g., level of development) and variation of methodologies and data sets. The impact of any particular parameter can be country specific. Therefore, country-specific further study is crucial for setting and executing correct policies.

Bangladesh was one of the poorest countries in the universe during the 1970s and 1980s, when the poverty level was high and unemployment was massive. It was known as a country of floods, cyclones and other natural calamities. Surprisingly, Bangladesh has largely overcome these problems. Its economic growth rate is remarkable, especially in recent years. In 2019, Bangladesh was ranked as the world’s seventh fastest-growing economy with a real GDP growth rate of 8.1%, which put it ahead of all Asian countries [21]. According to World Bank [22] report, over the past decade, Bangladesh remained in the list of the fastest-growing economies in the world. Even in the fiscal year 2021 (covid pandemic period), the real GDP growth rate was 6.9% while most of the countries experienced negative GDP growth. The poverty level also reduced dramatically. According to the Asian Development Bank report [23], in 2018, only 21.8% of the population were below the national poverty line. While the percentage of employed workers living below the poverty line was 73.5% in 2010, it dropped to 10.4% in 2018. The official unemployment rate was 4.29% in 2019, and the country improved considerably in health, education, infant mortality and life expectancy. It is argued that the rapid development of the Ready-Made Garments (RMG) industry, personal remittances and stable macroeconomic conditionsare the main contributory factors behind this growth [22]. The role of the Bangladesh government in planning and policy-making also contributed to positive GDP growth. Bangladesh Delta Plan 2100 focuses on economic growth [24]. Bangladesh’s 8^th^ Five-Year Plan (2021–2025) targeted 8.51% GDP growth and aimed to reduce the poverty rate to 15.6% at the end of the plan period. To achieve the target Bangladesh government is encouraging private investment by offering various incentives, taking measures for diversification and human capital development.

**Fig 1:**
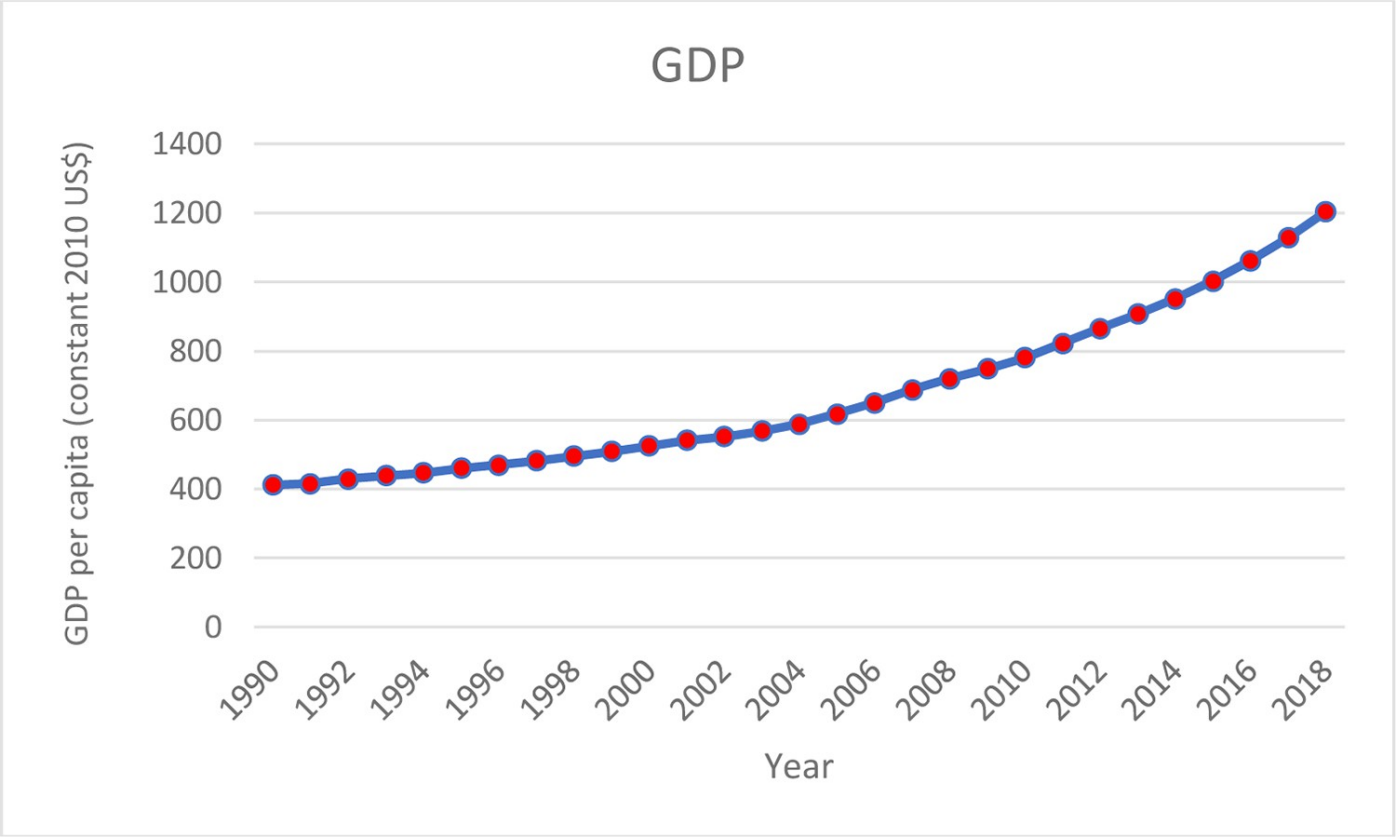
Trend of GDP per capita in Bangladesh from 1990 to 2018.

**Fig 2:**
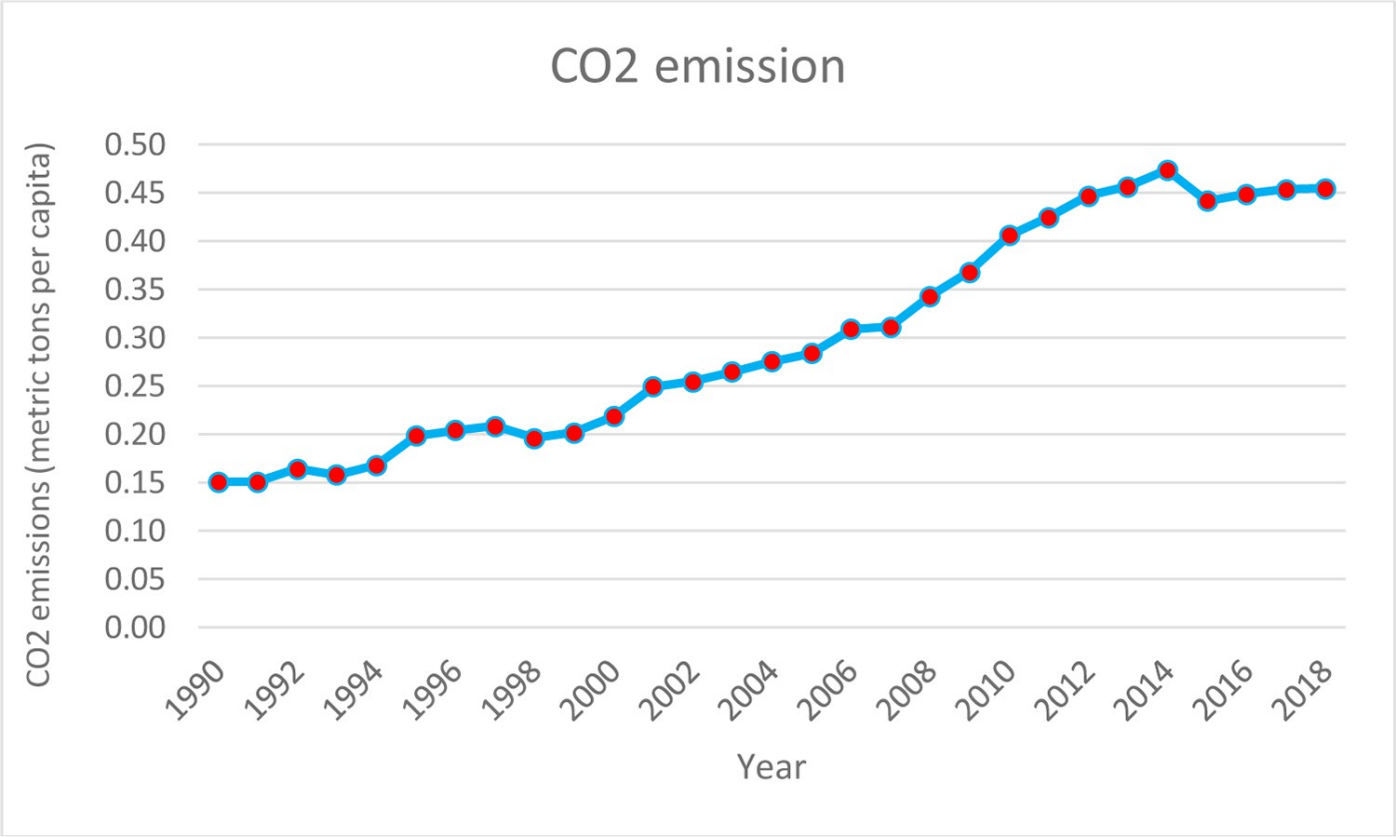
Trend of CO2 emissions in Bangladesh from 1990 to 2018.

**Fig 3:**
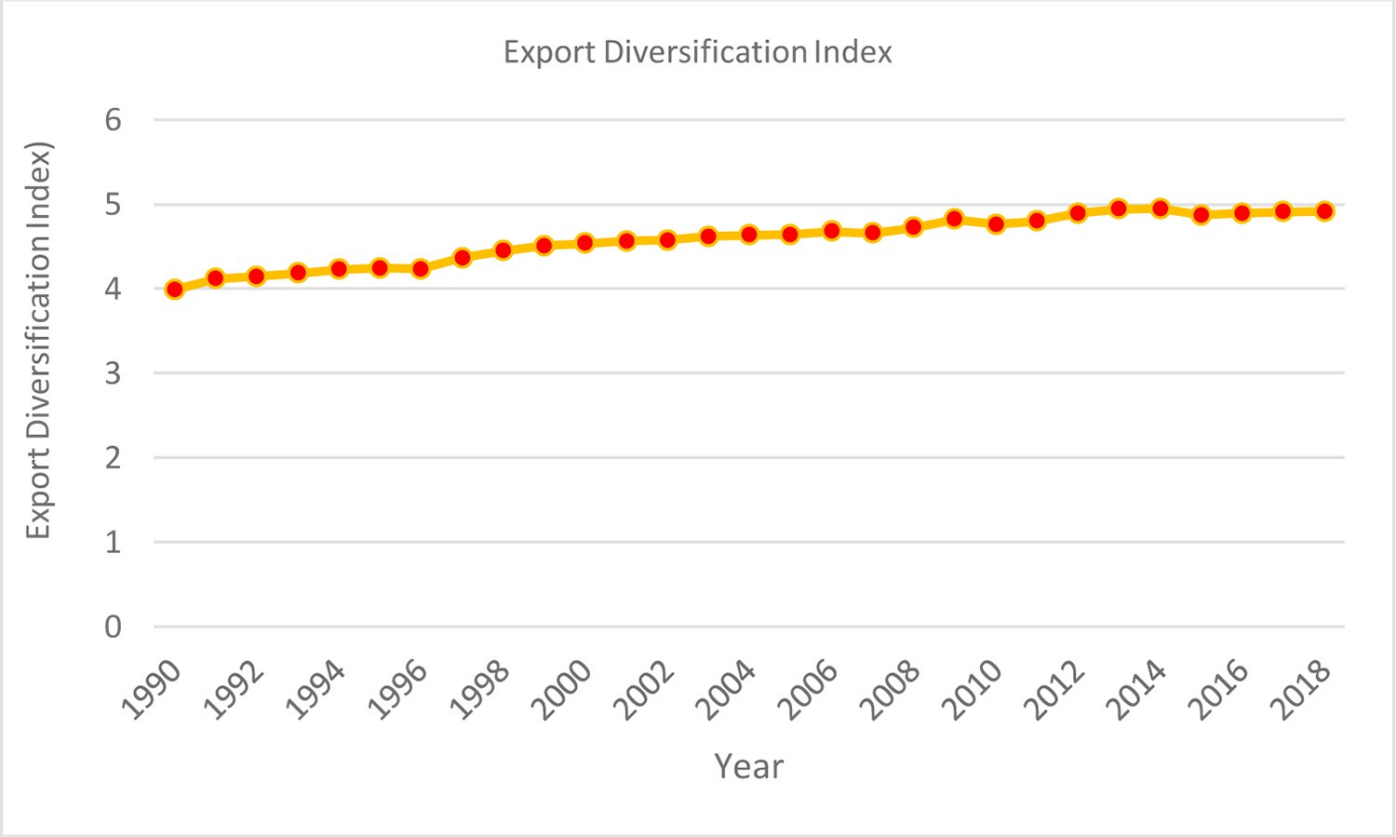
Trend of export diversification index in Bangladesh from 1990 to 2018.

The worrying fact is that Bangladesh’s export base is very narrow, limited to only certain products. The contribution of exports to GDP is only 14.80% against the import-GDP ratio of 23.44% [25]. RMG contributes 72% of total export earnings over the period 2016–2019. The country largely depends on the European Union and North American countries for RMG exports [26]. If these two leading economies are affected by any sort of uncertainty or economic downturn, Bangladesh’s RMG export would fall into a vulnerable condition. The current account deficit is observed almost every year; and in 2019–2020, it was US$ 4125 million [27]. Personal remittance, which is a component of the current account, is gradually decreasing. While remittance was 10.59% of GDP in 2012, this dropped to 5.7% in 2018 (World Bank, 2020). More importantly, Bangladesh is now ranked as the most contaminated country in the world with an average PM 2.5 concentration of 97.1 [25]. Considering all these facts, it is now a significant research question to explore the driving forces of the long-term economic growth of the country. Figs 1–3 show the trend lines of the main variables of interest.

It appears that GDP growth in Bangladesh is sustained over the period at a noteworthy level and is revealed monotonically very steady. The other two variables CO_2_ emissions and export growth are also increasing in the considered sample period very resolutely. So, all three variables are exhibiting similar moving gestures. Altogether it is very likely that they may have very close inter-relationships. Besides, it is perceptible that historical co-movement of the considered variables shows the possible association among them. In other words, it manifests that perhaps the variables have a causal linkage and one subset of the variable has the predictive capacity of the rest. Accordingly, it is palpable that as per the theoretical hypothesis if the past trend prevails unchanged, export concentration and income growth of Bangladesh will determine the future growth of carbon emissions level.

Therefore, this research aims to explore the driving forces of the economic growth of Bangladesh with particular attention to the role of environmental pollution, export concentration and remittances. Other considered variables are capital stock, labour force, consumption expenditure, and inflation rate. These variables are selected based on theoretical notions, data availability and past literature. For example, capital and labour are used following the neoclassical growth model; CO_2_ emissions are used following Rahman [16, 28, 29]; export concentration variable is used in line with [30–32]; remittance and inflation rate are used following [8]; and consumption expenditure is used observing [33, 34]. The major contributions of this research are as follows. First, this is the first-ever study in Bangladesh that examines the growth factors considering two often ignored but important variables, environmental problem and export concentration, along with other related variables. Hence, our study mitigates the misspecification and omitted variable biases. Second, our findings are reliable, as they are supported by the different diagnostic tests and robustness tests by FMOLS and CCR regressions. Third, our research is based on up-to-date and comprehensive reliable data; so the policymakers can derive benefits from the findings of this study to formulate and execute proper socio-economic and environmental policies.

## 2. Literature review

In the past, researchers tried to explore the various factors of economic growth. Here, we will review the past empirical literature under the following five strands, which are consistent with the theme of this research.

### 2.1. Economic growth–CO_2_ emissions nexus

The nexus between environmental pollution and economic growth is still unclear. One school of thought suggests that economic growth leads to an increase of pollution monotonically [35]. However, the second school of thought believes that economic growth helps to reduce pollution in the long-run through production efficiency. Economic development aids firms in enhancing energy efficiency, significantly cutting pollutant particle emissions. In addition, the establishment of expensive but green production units that are considered environmentally friendly can be installed due to having a higher income. There is a third-party belief that there exists a non-linear relationship economic growth and pollution. This relationship can be explained through the famous Environmental Kuznets Curve (EKC) hypothesis, which explains that at the initial level of economic development, CO2 emissions enhance with an increase of economic growth; once certain level of economic growth is achieved, the country can afford environmental friendly technology and then economic growth contributes to decrease emission level.

Empirically, there are mixed results on the relationship of both variables. For example, a bidirectional link between CO_2_ emissions and economic growth is revealed by [15] for South Asia [20], for five OPEC countries [36], for MENA countries, and [29] for Bangladesh. In contrast [16], observed a unidirectional causality running from economic growth to CO_2_ emissions in Asian populous countries. [37] found a neutral effect of CO2 emissions on the economic growth of Ghana during 1971–2018. While [15] found the positive effects of CO_2_ emissions on economic growth [38], have confirmed an inverted U-shaped linkconnection between these two variables for OECD countries. Furthermore [39], found no long-run linkage between these two variables for India.

### 2.2 Economic growth–Export diversification nexus

Theoretically, it is expected that export diversification (concentration) boosts (contracts) of exports, which in turn enhances (decreases) economic growth. However, there is also a lack of consensus on the relationship of economic growth and export diversification. While most of the studies reported the positive effects of export diversification on economic growth, a reverse effect was also found. The positive effects were found by the studies of [40] for 114 countries [31], for 24 small states [30], for a group of island countries and [41] for South Africa. In contrast [42], found no positive effect for less developed countries. [43, 44] also reached the same conclusion for a group of Middle Eastern countries, and 68 countries, respectively, and expressed the opinion that too much export diversification may be detrimental to the growth of the country. Further evidence by [45] says that in case of the USA both exports and export quality growths influence income growth positively, and income growth increases fossil fuel consumption.

With a panel data study [46], inspected three developing parts of the world: Sub-Saharan Africa, Latin America, and Asia, for the period 1995 to 2015, and made three subsamples in order to compare each region with the others. Their finding was that initially any sort of export diversification would have a positive influence on growth but subsequently in the second stage, transforming productive structure to more sophisticated products and more diversification would be required to make the growth trajectories sustainable.

### 2.3 Economic growth–remittance nexus

Theoretically, the effect of remittance on GDP is expected to be positive if the remittance can be used in the productive sector. Remittance can also increase aggregate demand which should boost economic growth. Studies on the relationship between economic growth and remittance are limited in the literature. These studies show diverse findings. For example, the studies of [8, 47–49] found positive influence of remittance on the economic growth of six European countries, 21 developing countries, Bangladesh and South Asian countries, respectively. On the other hand, negative effects were also revealed by the studies of [50, 51] for Bangladesh. [52, 53] considered that remittance contributed to growth positively if the countries were open, and the financial sector was underdeveloped. However, an IMF report [54] found no link between GDP growth and remittances in 101 developing countries for the period 1970–2003.

### 2.4 Economic growth–consumption expenditure nexus

[55] have noted that there is no concensus with regard to the direction of causality between consumption expenditure and economic growth. Both can work as cause and effect. A study by [56] found consumption to be the result, of growth for Bangladesh instead of cause. In contrast, a study by [57] found consumption to be the cause of growth for India, demonstrating a unidirectional causality from consumption expenditure to real economic growth. A study by [58] also found that GDP and consumption expenditure had a significant influence on each other in Bangladesh. The work of [59] also proved the long-run and short-run positive influences of consumption spending on economic growth in Indonesia. The study revealed that the increase in economic growth was 2.88% if the consumption spending rose by 1%. However [60], found no significant effect of consumption expenditure on the GDP of Nigeria. Lastly [61], found that income had no influence on long-run power consumption by households in 11 OECD countries.

### 2.5 Economic growth—Inflation nexus

The inflation rate is one of the leading factors of economic growth [4]. However, there is a lack of confirmed conclusion about the link between economic growth and the inflation rate; hence, the results are inconclusive. [62] identified a positive link between inflation and economic growth in a study of four South Asian countries; the same observation is also revealed by the studies of [63, 64] for Bangladesh and six South Asian countries, respectively. In contrast [65, 66], confirmed a statistically significant negative link, in the long run, between economic growth and inflation for Bangladesh and Qatar, respectively.

Further [67], found no significant long-run link between economic growth and inflation but discovered a short-run negative relationship in Turkey. In a separate study on Iran [68], identified a structural breakpoint effect and found that the maximum growth incurring threshold level of inflation rate was in between 9% and 12% meaning that if the inflation existed below and above this range there would be a positive and negative relationship between inflation and economic growth rates, respectively. If it existed within the range, the country would incur a maximum growth rate.

Clearly, the above literature indicate the inconclusiveness about the effects of noted variables on economic growth. Moreover, none of the studies had used all of our considered variables jointly which may lead to the wrong specification of the model due to the omission of important explanatory variables. This paper explains the joint impact of five explanatory variables, in addition to capital and labour, which we believe provides a robust model to explain the economic growth in Bangladesh. Additionally, this research has used two important variables: CO_2_ emissions and export diversification, which have been mostly unexplored by earlier papers in the case of Bangladesh. Export diversification should not be ignored because by doing so, the country could follow a direction leading to implementation of the wrong policies. This research contributes to filling up this gap as well.

## 3. Data, model and methodology

### 3.1 Data

This research employs annual time-series data covering 1990 to 2018 for Bangladesh. It comprises annual data defined as per capita economic growth constant 2010 US$, per capita gross capital formation (constant 2010 US$), per capita labour, per capita carbon emissions in metric tons, personal remittances (% of GDP), per capita consumption expenditure, inflation rate and export diversification index. All data except the export diversification index are downloaded from the World Development Indicator (WDI), World Bank. The data on export diversification are collected from the International Monetary Fund (IMF).

### 3.2 Theory and model specification

We model the relationship between our outcome variable (economic growth) and explanatory variables (capital, labour, CO_2_, remittance, consumption, inflation, export diversification) using a simple production function. The neo-classical growth theory is the basis for our empirical model. We employ Cobb-Douglas production function [69] as under:

$$y=k^{\beta 1}l^{\beta 2}\varepsilon^{\mu }$$ (1)

Where, y is output or economic growth; k and l are capital and labor, respectively, used in production.; εis the erorr term, which captures all other unobserved factors of production; β_1_, and β_2_ are the output elasticity related to capital and labor, respectively. This production function can be extended by adding some other relevant variables, which is shown in Eq (2) below. by adding other variables.


$$y=k^{\beta 1}l^{\beta 2}co2^{\beta 3}rem^{\beta 4}con^{\beta 5}inf^{\beta 6}edi^{\beta 7}\varepsilon^{\mu }$$ (2)

Where β_1,_ β_2,_ β_3,_ β_4,_ β_5,_ β_6_ and β_7_ are representations for the elasticity of capital, labour, carbon emission, remittance, consumption, inflation, export diversification. Following previous studies [70, 71], we adopt the following semi-elasticity model of the long-run link of the variables:

$$lny_{t}=\beta_{0}+\beta_{1}lnk_{t}+\beta_{2}l_{t}+\beta_{3}co2_{t}+\beta_{4}rem_{t}+\beta_{5}lncon_{t}+\beta_{6}inf_{t}+\beta_{7}edi_{t+}\varepsilon_{t}$$ (3)

For our empirical study, we have used model (3), where y, k, l, co2, rem, con, inf and edi represent, respectively, per capita economic growth, per capita capital formation, per capita labour force, per capita carbon emissions, personal remittances, per capita consumption, inflation and export diversification index in Bangladesh. ln, t and ε are natural logarithms, time and error terms, respectively. The parameters β_1_ β_2_, β_3_, β_4_, β_5_, β_6,_and β_7_ indicate the long-run elasticity estimates of capital formation, labour, CO_2_ emissions, personal remittances, consumption, inflation and export diversification, respectively.

### 3.3 Unit root tests

This study uses three-unit root tests, Augmented Dickey-Fuller [ADF] [72] and Phillips and Perron [PP] [73], to check for the integration order of variables. The Fisher test of unit root using ADF estimates the regression equation as follows:

$$\Delta Y_{t}=\alpha +\beta_{t}+\theta y_{t-1}+\sum_{j=1}^{n}\mu_{i}\Delta Y_{t-1}+e_{t}$$ (4)

where α, β, n, μ, and e indicate the intercept, the coefficient on the time trend T, number of lags, the coefficient on the lagged dependent variable, and random error, respectively.

[73] suggest the following unit root test.


$$\Delta Y_{t}=\alpha +\beta_{t}+\theta y_{t-1}+e_{t}$$ (5)

### 3.4 ARDL (Autoregressive distributed lag) co-integration analysis

The existing literature provides several econometric techniques: Johansen and Juselius [74] Engle and Granger [75], and Johansen [76] tests for a co-integration relationship. This study incorporates the ARDL-bounds testing approach to test the long-run co-integration link, which was originally developed by [77] and further developed by [78]. The ARDL co-integration method is superior in comparison with the other co-integration approaches mentioned above. Firstly, this method tests the long-run link between variables whether the underlying repressors are integrated of order I(0), I(1) or fractionally integrated. Secondly, the ARDL test is an efficient estimator even if the sample size is not large. Thirdly, this method gives unbiased estimates of the long-run model and effective statistics even if some of the repressors are endogenous. Fourthly, these techniques estimate long and short-run parameters at the same time. Finally, this test incorporates a dynamic error correction model (ECM), which can be inferred by a linear transformation.

$${\Delta lny}_{t}=\theta_{0}+\sum_{i=1}^{p}\theta_{1i}\Delta {lny}_{t-1}+\sum_{i=1}^{p}\theta_{2i}\Delta {lnk}_{t-1}+\sum_{i=1}^{p}\theta_{3i}\Delta l_{t-1}+\sum_{i=1}^{p}{\theta_{4i}\Delta co2}_{t-1}+\sum_{i=1}^{p}\theta_{5i}\Delta {rem}_{t-1}+\sum_{i=1}^{p}{\theta_{6i}\Delta con}_{t-1}+\sum_{i=1}^{p}{\theta_{7i}\Delta inf}_{t-1}+\sum_{i=1}^{p}\theta_{8i}\Delta {edi}_{t-1}+\theta_{9}{lny}_{t-1}+\theta_{10}{lnk}_{t-1}+\theta_{11}l_{t-1}+\theta_{12}{co2}_{t-1}+\theta_{13}{rem}_{t-1}+\theta_{14}{con}_{t-1}+\theta_{15}{inf}_{t-1}+\theta_{16}{edi}_{t-1}+{\theta \varepsilon }_{t}$$ (6)

where Δ is the first difference. ε is the white noise error term. p is the optimal lag length grounded on the Bayesian Information Criterion (BIC) and Akaike Information Criterion (AIC). The existence of a long-runlink among economic growth, capital formation, labour, carbon emissions, personal remittances, consumption, inflation and the export diversification index in Bangladesh is based on F-statistic that tests the null hypothesis (H_0_: *θ*_9_ = *θ*_10_ = *θ*_11_ = *θ*_12_ = *θ*_13_ = *θ*_14_ = *θ*_15_ = *θ*_16_ = 0) of having no co-integration among the variables in Eq (5) against the alternative hypothesis (H_1_: *θ*_9_ ≠*θ*_10_ ≠*θ*_11_ ≠*θ*_12_ ≠*θ*_13_ ≠*θ*_14_ ≠*θ*_15_≠*θ*_16_ ≠0). This study adopts the critical values of [78]. Our calculated F-statistic and critical values can be interpreted based on three assumptions: First, if the calculated F-statistic is higher than the upper critical bound, the null hypothesis will be rejected. Second, if the calculated F-statistic lower than the upper critical bound value, the null hypothesis will be accepted. Finally, if the calculated F-statistic value is within lower and upper critical bound, then the result is considered inconclusive.

## 4. Results and discussion

### 4.1 Summary statistics

Table 1 provides descriptive statistics. Individual variables are defined as follows:

lny = logarithmic value of yearly per capita constant GDP,

lnk = logarithmic value of yearly per capita capital stock formation,

l = annual per capita labour,

co2 = annual per capita co2 emission in metric tons,

rem = yearly foreign personal remittance inflow as a percentage of GDP,

lncon = logarithmic value of yearly per capita gross consumption expenditures,

inf = year on year inflation rate, and

edi = export diversification index.

**Table 1:** Descriptive statistics.

	lny	lnk	l	co2	rem	lncon	inf	edi
Mean	6.456	4.942	0.370	0.299	5.802	4.726	5.779	4.581
Median	6.377	4.894	0.372	0.275	5.407	4.656	5.816	4.632
Maximum	7.093	5.923	0.425	0.474	10.588	5.663	19.143	4.951
Minimum	6.019	4.114	0.328	0.151	2.465	3.940	0.156	3.990
Std. Dev.	0.329	0.561	0.026	0.114	2.707	0.524	3.182	0.286
Skewness	0.409	0.114	0.156	0.245	0.377	0.154	2.401	-0.462
Kurtosis	1.917	1.814	2.259	1.546	1.691	1.796	12.013	2.031
Jarque-Bera	2.225	1.762	0.780	2.843	2.758	1.865	126.007	2.164
Probability	0.329	0.414	0.677	0.241	0.252	0.394	0.000	0.339

### 4.2 Analysis of unit root tests

The results of unit root tests are noted in Table 2 below.

**Table 2:** Unit root analysis.

	Augmented Dickey-Fuller test statistic		Phillips Pearson test statistic	
	Level	1^st^ difference	Level	1^st^ difference
y	0.391	-3.478**	0.009	-3.579***
k	-1.581	-5.832***	-3.140	-5.748***
l	-1.428	-0.058	-1.265	-5.274***
co2	-1.769	-2.476	-1.835	-4.309**
rem	-0.883	-2.789	-0.295	3.654**
con	-1.059	-4.535***	-1.586	-4.912***
inf	-3.301*	-4.516***	-4.234*	-7.319***
edi	-0.958	-3.547**	-2.094	-5.679***

Note: *, ** and *** denote 10%, 5% and 1% levels of significance, respectively. All variables are testes with intercept and trend.

### 4.3 Selection criteria for lag length

The lag order of the variables is vital for ARDL model specification. The ARDL is based on the number of regressions (P +1)^K^ where P and K indicate the number of maximum lags and the number of variables in each variable respectively. The appropriate lag selection procedure is based on the Akaike information criterion (AIC), Hannan-Quinn information criterion (HQ), the sequential modified LR test statistic (LR), Schwarz information criterion (SC) and final prediction error (FPE). Table 3 shows the lag length criterion selection. We use AIC criteria to identify appropriate lag lengths. Following [79–81] we select AIC criteria for this study.

**Table 3:** Selection criteria for lag length.

Lag	LogL	LR	FPE	AIC	SC	HQ
0	80.31983	NA	0.000184	-5.785587	-5.395546	-5.677406
1	101.4195	27.00758	3.73e-05	-7.393560	-6.954765	-7.271857
2	104.6789	3.911331*	3.15e-05*	-7.574316*	-7.086765*	-7.439090*
3	104.8181	0.155881	3.44e-05	-7.505450	-6.969145	-7.356702
4	104.9543	0.141612	3.76e-05	-7.436343	-6.851283	-7.274072

* indicates lag order selected by the criterion

LR: sequential modified LR test statistic (each test at 5% level)

FPE: Final prediction error

AIC: Akaike information criterion

SC: Schwarz information criterion

HQ: Hannan-Quinn information criterion

### 4.4 Analysis of co-integration tests

The next step is to estimate the long-run link through the ARDL bound test technique. Following [79–81], this study choses the AIC criteria to identify correct lag length. The results on the selection of lag length provided in Table 3 indicate that the optimal lag length is 2.

Table 4 reports the F-statistic of co-integration among the variables for the long-run nexus. Our calculated F-statistic is higher than the upper critical value, which rejects the null hypothesis of no co-integration. So, the ARDL bound F-statistic for a long-run co-integration test provides evidence of the long-run nexus between, capital formation, carbon emissions, personal remittance, consumption, inflation, export diversification and economic growth at 1% significance level in Bangladesh.

**Table 4:** The results of the ARDL bounds test for co-integration.

lny = f(lnk, l, co2, rem, lncon, inf, edi)			
F-statistic	5.895***		
Critical values	1%	5%	10%
Lowe bound I(0)	3.599	2.597	2.196
Upper bound I(I)	5.230	3.907	3.337

Notes: *** indicates 1% statistical significance. The critical values are achieved from Pesaran et al. (2001).

### 4.5 Analysis of long and short-run estimates

Table 5 reports the results of the long-run coefficients of ARDL co-integration estimates. The relation between economic growth and CO_2_ emissions is positive and significant at 5%level. We find that there is a statistically significant positive sign with a magnitude of 0.308. If CO_2_ emissions (metric tons per capita) are increased by one unit, we expect the actual GDP per capita to increase by 30.8%. The positive link between CO_2_ emissions and economic growth may be due to high industrial activities, which emit more carbon in the environment in Bangladesh. This finding is consistent with the results of [15]. Hence, Bangladesh would need to find alternative energy sources to control carbon emissions. The coefficients of personal remittance are negative and significant, indicating that an increase in the personal remittance to GDP ratio results in a decrease in economic growth. This finding is contrary to the findings of [8, 49] but in line with the results of [50, 51]. The reason for negative relationship of this variable may be due to the fact that most of the remittances are spent in unproductive channels (e.g. meeting basic consumption needs of families) rather than productive channels like investment. The link between consumption expenditure and economic growth is positive at 10% significant level, and is similar to the findings of [59]. Since aggregate consumption is a fundamental component of GDP, this positive relationship is rational. A negative and significant effect of inflation on economic growth in the long-run is found, which implies that inflation is a threat to economic growth in Bangladesh. This observation supports the result of [66] but contradicts the findings of [63]. Inflation reduces the purchasing power of people, and consumers’ confidence reduces, which negatively affect economic growth. So, negative effects of inflation on economic growth of Bangladesh is theoretically consistent. Finally, the impact of export diversification index on economic growth is significantly negative. If export diversification index is increased by one unit, the actual GDP per capita will decrease by 37.4%. It is noted that the higher export diversification index indicates lower export diversification (export concentration), a measure that adversely affects economic growth in Bangladesh. In other words, the impact of export concentration on the economic growth of Bangladesh is negative, indicating that export concentration is a major threat to long-term economic growth in Bangladesh. This result is justified as a country’s dependence only on a few exportable items is always risky due to volatile global economic conditions. This finding reaffirms the observations of [31, 40, 82].

**Table 5:** The long-run ARDL cointegrating model (2, 2, 1, 0, 0, 2, 2, 2).

Variable	Coefficient	Std. Error	t-Statistic	Prob.
lnk	0.319	0.282	1.130	0.291
l	-0.931	0.729	-1.278	0.237
co2	0.308	0.102	3.034**	0.016
rem	-0.007	0.002	-3.547***	0.008
lncon	0.502	0.255	1.969*	0.085
lnf	-0.005	0.001	-3.993***	0.004
edi	-0.374	0.083	-4.493***	0.002

Notes: _*, **_ and _***_ indicates statistical significance at 10%, 5% and 1%, respectively. The maximum lag to be used four. The optimal lag structure is chosen by Akaike Information Criterion.

The results of the short-run estimated of ARDL co-integration error correction are also reported in Table 6. The short-run coefficient of first lag of the first difference of economic growth is positive at 1% significant level implying that there is a dynamic relationship between variables. The short-run coefficients of first difference and first lag of the first difference of capital formation are -0.197 and -0.473; these are significantly negative. The short-run coefficients of first difference and first lag of the first difference of CO2 emissions are 0.341 and 0.207; these are positive and statistically significant. Interestingly, the coefficients of first difference and first lag of the first difference of the inflation are negative and positive respectively and significant at 1% level. The short-run coefficients of first difference and first lag of the first difference of export diversification are -0.145 and 0.104; these are negative and positive, respectively and statistically significant. The elasticity coefficient of lagged error correction term, ect_t-1_, is -0.820 and statistically significant, indicating that the deviation of variables is adjusted by 82% per year from the short-run to long-run equilibrium.

**Table 6:** The ARDL cointegrating short-term error-correction model (2, 2, 1, 0, 0, 2, 2, 2).

Variable	Coefficient	Std. Error	t-Statistic	Prob.
Constant	3.749	0.412	9.107***	0.000
Δlny(-1)	0.730	0.071	10.344***	0.000
Δlnk	-0.197	0.080	-2.472**	0.039
Δlnk(-1)	-0.473	0.093	-5.068***	0.001
Δl	0.062	0.254	0.244	0.813
Δcon	0.341	0.058	5.933***	0.000
Δcon (-1)	0.207	0.078	2.649**	0.029
Δinf	-0.002	0.000	-5.607***	0.001
Δinf(-1)	0.001	0.000	2.698**	0.027
Δedi	-0.145	0.021	-7.040***	0.000
Δedi(-1)	0.104	0.023	4.459***	0.002
ect(-1)	-0.820	0.090	-9.068***	0.000

Notes: _*, **_ and _***_ indicates statistical significance, respectively, at 10%, 5% and 1%. The maximum lag to be used four. The optimal lag structure is based on Akaike Information Criterion.

### 4.6 Diagnostics tests

This study uses several diagnostic tests to ensure the stability of the model. The results of diagnostic tests from ARDL estimates are shown in Table 7. The R-squared and adjusted R-squared are 0.999 and 0.997, respectively confirming that the model explains about 99% variation in the dependent variable. The DW statistic is 2.810, which implies that the model is not spurious. The LM test indicates that there is no serial correlation. The normality test implies that the error terms are distributed normally. The heteroscedasticity test equals 0.578. Overall, the model passes all the diagnostic tests with a good fit.

**Table 7:** Diagnostics tests.

R squared	0.999
Adjusted R squared	0.997
F-statistics	7134 (0.000)
Durbin Watson test	2.810
Breusch-Godfrey serial correlation LM test	3.483 (0.099)
Jarque-Bera normality test	0.733(0.693)
Breusch-Pagan-Godfrey heteroskedasticity test	0.578 (0.841)

**Fig 4:**
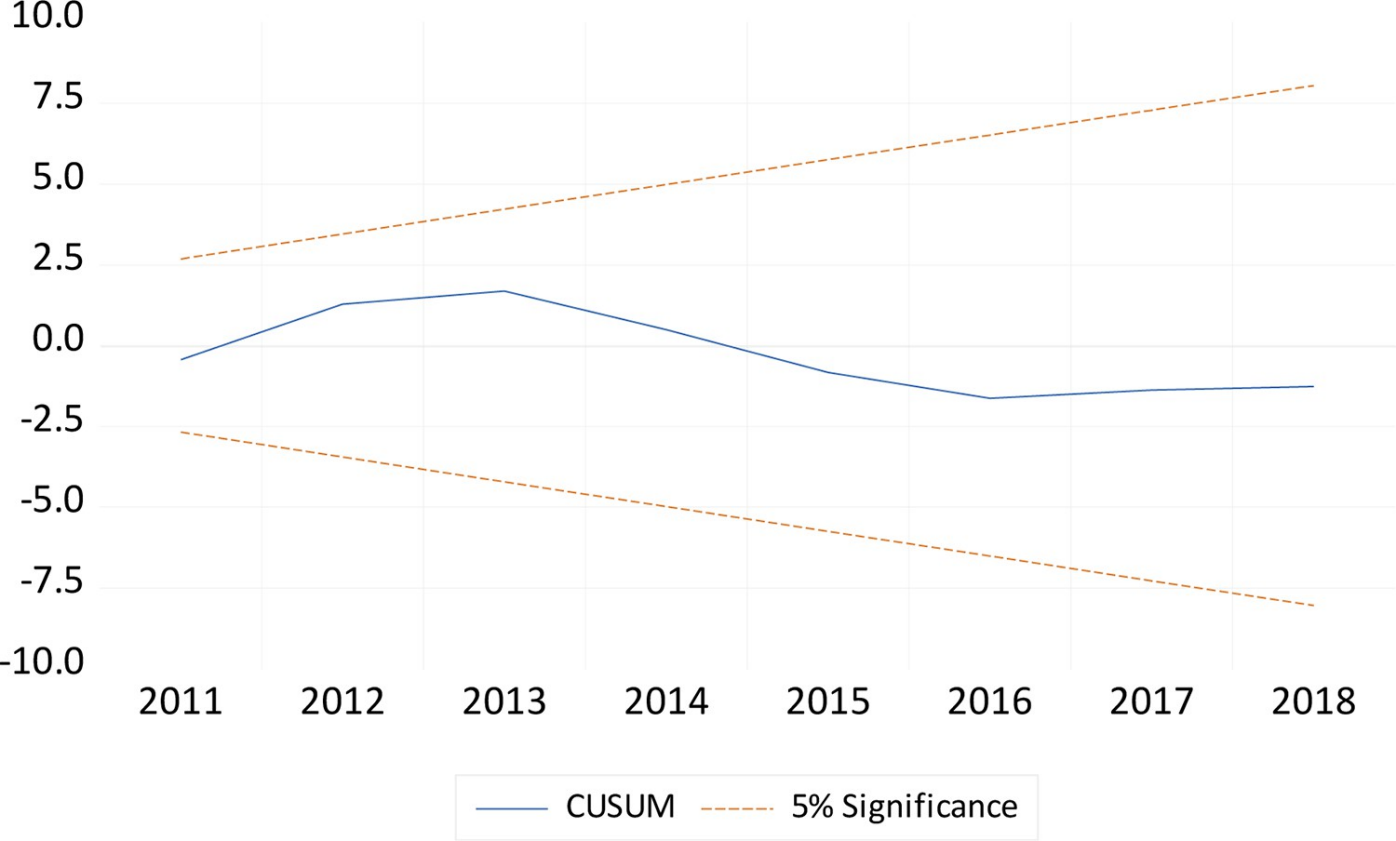
Plot of the cumulative sum of recursive residuals (CUSUM).

**Fig 5:**
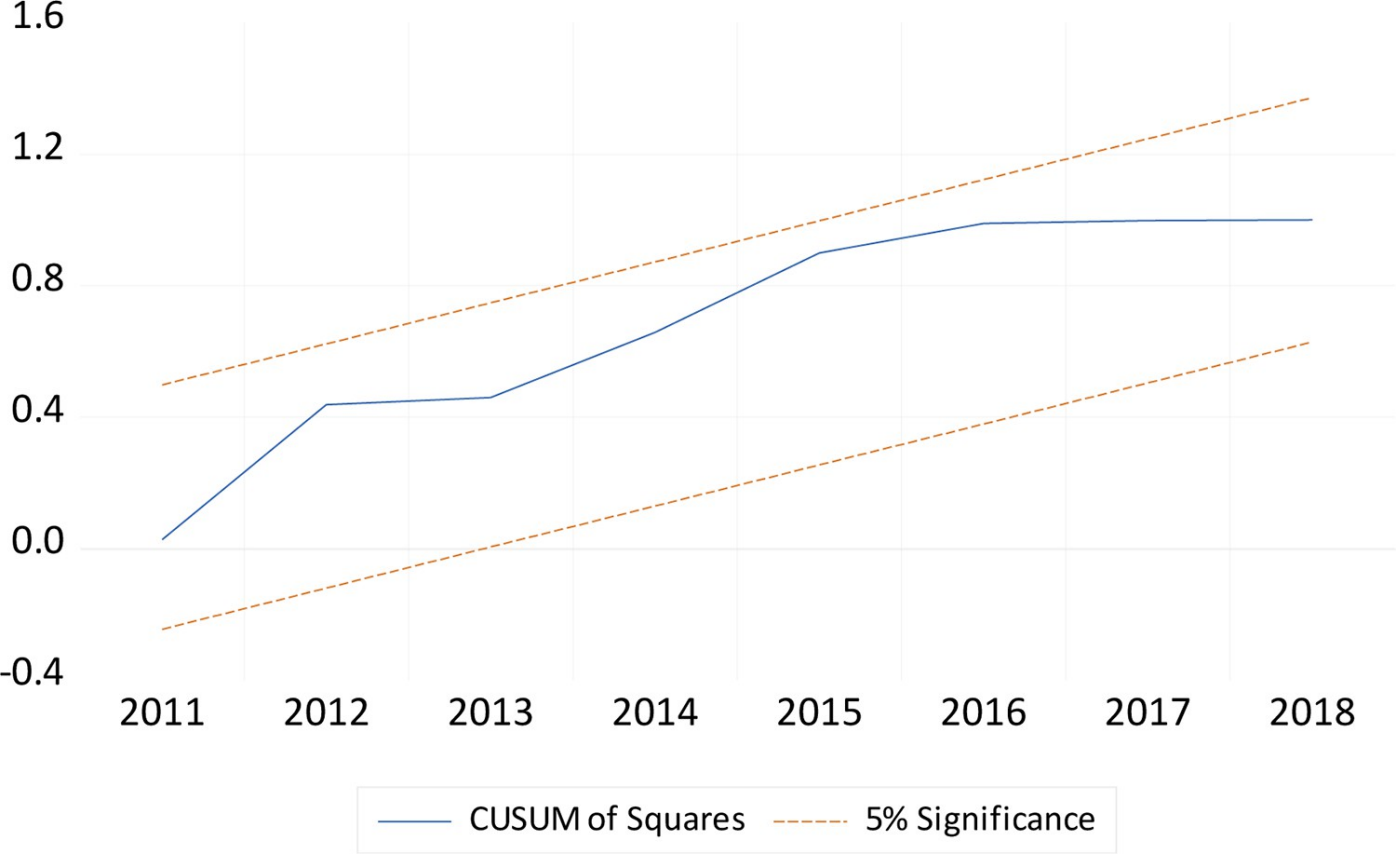
Plot of the cumulative sum of recursive residuals of squares (CUSUMSQ).

The cumulative sum of recursive (CUSUM) and cumulative sum of recursive residuals of squares (CUSUMSQ) tests are performed to test the stability of the parameters. The results are shown in Figs 4 and 5.

Figs 1 and 2 present the stability of the long-run and short-run estimates by graphical plots of CUSUM and CUSUM of Squares. The figures show that estimated coefficients exist within the upper and lower critical bounds at the 5% significance level, suggesting that the estimated parameters are stable over the periods.

We used two additional tests of Fully Modified Least Squares (FMOLS) and Canonical co-integrating regression (CCR) to confirm the robustness of our long-run coefficients from ARDL. Previous studies confirm that FMOLS is a superior estimate, as it is free from small sample size bias, endogeneity and serial correlation [70, 83, 84]. Tables 8 and 9 reports the results from FMOLS and CCR. Both estimates are consistent with the ARDL results.

**Table 8:** Fully Modified Least Squares (FMOLS).

Variable	Coefficient	Std. Error	t-Statistic	Prob.
lnk	0.261	0.165	1.586	0.129
l	-0.466	0.732	-0.637	0.531
co2	0.513	0.130	3.954***	0.001
rem	-0.007	0.002	-3.596***	0.002
con	0.461	0.157	2.931***	0.008
inf	-0.005	0.001	-6.734***	0.000
edi	-0.327	0.049	-6.621***	0.000
Constant	4.570	0.173	26.392***	0.000

Notes: _*, **_ and _***_ indicates 10%, 5% and 1% statistical significance, respectively.

**Table 9:** Canonical co-integrating regression (CCR).

Variable	Coefficient	Std. Error	t-Statistic	Prob.
lnk	0.256	0.148	1.731*	0.099
l	-0.712	1.190	-0.598	0.556
co2	0.493	0.165	2.996***	0.007
rem	-0.007	0.002	-3.367***	0.003
con	0.473	0.142	3.318***	0.003
inf	-0.004	0.001	-4.421***	0.000
edi	-0.309	0.040	-7.690***	0.000
Constant	4.551	0.132	34.354***	0.000

Notes: _*, **_ and _***_ indicates statistical significance at 10%, 5% and 1%, respectively.

## 5. Conclusion and policy implications

Bangladesh is now the fastest growing country in Asia. This study investigated the driving forces behind the phenomenon of the economic growth of this emerging country. In particular, attention was paid to the roles of export concentration, environmental pollution and personal remittances received. Using annual data over the period of 1990–2018, the study employed the ARDL bound testing approach along with other sophisticated econometric approaches and diagnostic tests for the robust findings. The obtained results confirm that export concentration, environmental pollution and high inflation are the major threats for the long-run sustainable growth in the country. The slow growth of the personal remittance earnings also affects economic growth negatively, as these are mainly used for unproductive household consumption. Consumption expenditure is positively associated with the economic growth.

On the basis of the study findings, some important policy implications can be drawn. First, economic growth is increasing because of extensive industrial production activities that require higher energy consumption. Current fossil fuel energy consumption is around 74%, which is the main source of CO_2_ emissions. Therefore, Bangladesh must increase the share of renewable energy consumption looking for alternative energy sources to curb environmental pollution. Second, in recent years, the remittance inflow is showing a downward trend; more importantly, there is no proper policy to increase the inflow and right use of the remittance in the productive sectors. Policymakers should take proper initiatives to enhance remittances and policy must be undertaken to channel the remittances for the use of investment in the country rather than household consumption. The current government initiative of 2% bonus on remittance inflow should continue; further incentives should be provided on the investment of the remittances. Third, Bangladesh’s narrow export base, which is highly dependent on the RMG now, must be expanded. Export diversification is essential for sustainable long-term growth. Proper market research for new exportable items must be pursued; export fares through foreign missions will be helpful to increase foreign demand for Bangladesh’s products. Initiatives in this regard should be jointly undertaken by the government and the private sector. Fourth, inflation should be kept within an acceptable range. Contractionary fiscal and monetary policies should be undertaken in such a way that desired aggregate consumption expenditure is maintained to increase the much-needed economic growth and living standards of the people.
